# Indiana State Dental Association

**Published:** 1868-07

**Authors:** 


### INDIANA STATE DENTAL ASSOCIATION.

The regular annual meeting of this society was held in Indianapolis on Tuesday, Wednesday and Thursday, commencing June 30th. There was a full attendance of the members, and a goodly number of visitors present.

There was much interest in the proceedings. The discussions were well calculated to elicit thought and investigation; there was manifested the clearest evidence that there is still rapid progress, especially in the science of our profession. Formerly, the art of our profession outstriped the science; but it is pleasing to know that the latter is coming up, and will soon be equal to, if not in advance of it. A thorough knowledge of the principles upon which the practice of our profession is based should be obtained before the practice is taken up, and we are glad to see everywhere indications of this.

This society is made up of the best men in the State—men who have made their impress, wherever they have moved in the profession. This society has exercised a very decided influence for good in the profession in Indiana.

The proceedings of this meeting will be published in the next number of the REGISTER.

